# Acute Oak Decline-Associated Bacteria: An Emerging Worldwide Threat to Forests

**DOI:** 10.3390/microorganisms13051127

**Published:** 2025-05-14

**Authors:** Alessandro Bene, Marzia Vergine, Giambattista Carluccio, Letizia Portaccio, Angelo Giovanni Delle Donne, Luigi De Bellis, Andrea Luvisi

**Affiliations:** 1 Department of Biological and Environmental Sciences and Technologies, University of Salento, 73100 Lecce, Italy; alessandro.bene@unisalento.it (A.B.); giambattista.carluccio@unisalento.it (G.C.); letizia.portaccio@unisalento.it (L.P.); andrea.luvisi@unisalento.it (A.L.); 2 Department of Agriculture, Rural Development and Environment, Section Osservatorio Fitosanitario, 70125 Bari, Italy; a.delledonne@regione.puglia.it; 3 National Biodiversity Future Center, 90133 Palermo, Italy

**Keywords:** acute oak decline, *Brenneria goodwinii*, *Gibbsiella quercinecans*, *Quercus* spp.

## Abstract

Acute oak decline (AOD) is a multifactorial disease that affects European oaks and represents a growing threat to forests. The disease results from a complex interaction between biotic and abiotic factors: the various environmental stresses, which vary depending on the area in question, and generally increased by climate change, predispose trees to attack by opportunistic pathogens. Among them, we focused on a bacterial consortium associated with AOD, consisting mainly of *Brenneria goodwinii*, *Gibbsiella quercinecans*, *Rahnella victoriana*, and *Lonsdalea britannica*, which produce degrading enzymes that contribute to phloem necrosis and the development of stem bleeds and bark cracks. However, the role of other pathogens, such as fungi, cannot be ruled out, but instead could be contributory. The potential involvement of xylophagous insects is also being studied, particularly *Agrilus biguttatus*, which, although, frequently associated with the disease, has not been conclusively demonstrated to act as an active vector of the bacteria. Currently, disease management requires integrated approaches, including monitoring and other forestry strategies to increase forest resilience. Given the phenomenon’s complexity and the risk of the future expansion of that bacterial consortium, further research is necessary to understand the dynamics and to develop effective containment strategies of AOD-associated bacteria.

## 1. Introduction

Oak forests are particularly recognized worldwide for their economic value and biodiversity [1]. In recent decades, however, such landscapes have been marked by several important tree diseases, including chestnut blight and ash dieback. These diseases are often the result of complex interactions between host, environment, pests, and pathogens [2]. In particular, oak forests have experienced substantial degradation due to the spread of oak decline across Europe [3]. Notably, the incidence of AOD-associated bacteria appears to be progressively expanding further south [4]. Oak decline appears to be becoming a global problem, as it has also been found in Japan [5] and North America [6]. Oak decline, recognized as a complex phenomenon, is one of the most significant forest syndromes worldwide. It is more difficult to understand and manage than the decline of other tree species due to the broad spectrum of oak species affected, including *Quercus cerris* L., *Q. frainetto* Ten., *Q. petraea* (Matt.) Liebl., *Q. pubescens* Willd., *Q. robur* L., and *Q. suber* L., as well as the different environmental conditions within their geographic range that may or may not favor the syndrome [7]. Indeed, the syndrome is considered a multifactorial network [8], with predisposing abiotic factors playing a crucial role. However, biotic factors, including subcortical insects and bacterial and fungal pathogens, also contribute to accelerating the decline process [9].

Two forms of oak decline are currently distinguished: “chronic oak decline,” which progresses gradually, and “acute oak decline,” which manifests itself more rapidly. Trees affected by chronic oak decline often show a gradual progression of symptoms over decades; in contrast, AOD is of greater concern due to its rapid development, causing significant tree mortality [10,11], although some individuals are able to survive [12]. Since 2006, outbreaks of AOD have been reported in England, affecting both native oak species, *Q. robur* and *Q. petraea* [13]. The syndrome, identified in Northern and Central Europe [14], has distinct symptoms and poses a significant threat to the world’s forests. However, the full range of causal factors remains poorly understood [15]. As mentioned, AOD is a complex syndrome, as it involves abiotic and biotic factors: abiotic predisposing elements, such as stresses related to variations in temperature and precipitation regimes across different areas, contribute to tree vulnerability, while biotic agents, including the beetle *Agrilus biguttatus* [16] and a range of bacterial species, mainly *Brenneria goodwinii*, *Gibbsiella quercinecans*, *Rahnella victoriana*, and *Lonsdalea britannica*, act as predominant factors in the fate of affected oak trees [17]. These different contributing factors can be divided into three categories: “predisposing” agents, which reduce a tree’s resilience in coping with stressors; “inciting” agents, which initiate visible symptoms of decline; and “contributing” agents, which promote disease progression [18]. Among these, stress resulting from a prolonged state of drought is a significant factor in oak decline, often linked to site-specific soil conditions [4]. It cannot be overlooked that this condition is also facilitated by climate change, as progressively warmer years and reduced rainfall contribute to oak susceptibility [19].

Specifically, the crucial involvement in the progression of AOD by different bacterial species is recognizable by specific external symptomatology, including longitudinal fissures and necrotic bark lesions (5–10 cm in size) exuding dark, sticky exudates [20]. As a result, the crown volume is drastically reduced, negatively impacting the tree’s entire fitness, which risks death within four to five years after the onset of symptoms [10]. In addition, characteristic “D”-shaped exit holes, associated with *A. biguttatus* evasion, are often observed on the stems of affected trees [21]. However, the understanding of the epidemiology of this disease, the routes of spread of the bacteria, and the specific sequence of events leading to the observed symptoms is still limited [22].

## 2. Role of Bacteria in AOD

The biotic variables that influence the development of AOD continue to be investigated. It is clear from the different necrotic tissues that these are associated with a complex poly-microbial community [23]. As mentioned before, consistently detected bacterial species include *G. quercinecans*, *B. goodwinii*, and *R. victoriana*, with *L. britannica* occasionally present [17,24] (Table 1). Identifying these species accurately is difficult due to their similarity in colony appearance, phenotypic traits, and 16S rRNA gene sequences. However, the most widespread bacterial families identified in symptomatic tissues include *Pseudomonadaceae*, *Enterobacteriaceae*, *Halomonadaceae*, *Shewanellaceae*, and *Acholeplasmataceae*. The diversity within this bacterial community appears to vary depending on the condition of the tissue and the presence of *A. biguttatus* galleries [22]. *G. quercinecans* and *B. goodwinii* are considered to be the two leading bacterial species responsible for AOD, as they are constantly detected in necrotic tissues and their exudates [13]. The genus *Brenneria* represents a distinct group of Gram-negative bacteria characterized by perithecial flagella [25]. In particular, *B. goodwinii* is an optional anaerobe [20,26] commonly associated with necrotrophic behavior, killing host tissues and using decomposing material as a substrate. Similarly, the genus *Gibbsiella* consists of Gram-negative, optional anaerobic bacteria [18]. These cells can exist individually, in pairs, or in groups of four and are characterized by thin fimbriae but not flagella [27]. Both *B. goodwinii* and *G. quercinecans* contribute to tissue necrosis and, with *A. biguttatus*, are responsible for severe cortical symptoms [28]. These bacteria are characterized by virulence genes commonly found in several plant pathogens, which are confirmed in the pathogenesis of AOD [23,29], and that allows them to pass from harmless commensal to aggressive necrotrophic pathogens. This transition is promulgated by the secretion of enzymes that degrade the cell wall polysaccharides of plants, such as pectinases, cellulases, and tannins [30].

It has also been verified that *G. quercinecans* prefers sugar metabolites from phloem tissue, while *B. goodwinii* tends to use oak sapwood as a carbon source [2]. When several pathogenic bacteria species are present together, the extent of necrosis is even more significant, suggesting a cumulative effect and potential synergistic interactions. In addition, the specific functions of *R. victoriana*, as well as other members of this microbiome within lesions, remain largely unclear, as do their interactions with the host and possible vectors [31].

*L. britannica* has shown different levels of pathogenicity, sometimes exhibiting virulence, but it is not always identified in AOD lesions, unlike the bacteria described above [23]. In fact, the degree of pathogenicity of these bacteria is not always predictable since it depends on their concentration and the overall health of the host oak [32]. Gathercole et al. [17] provided evidence of their presence in leaf and foliar tissues, suggesting that these bacteria are ubiquitous. Therefore, their presence in a single tree does not necessarily entail symptoms of AOD. However, the understanding of the ecological and environmental reservoirs that house these agents, both bacterial and non-bacterial, remains limited. Rainwater and forest soils are known to play this role in several plant pathogens. Pettifor et al. [33] describe *G. quercinecans* as a generalist within forest ecosystems, capable of surviving in both rainwater and soil. Meanwhile, *B. goodwinii* is a specialized endosymbiont of oaks, which does not survive outside its host.

On the other hand, the influence of fungal sepsis should not be underestimated. Fungi, particularly of the genera *Ceratocystis* and *Ophiostoma*, exploit oak trees weakened by environmental stress and insect attack to establish pathogenic relationships, thus triggering further vascular diseases, limiting the tree’s ability to absorb nutrients [34]. The same authors report a case where *B. goodwinii* was identified as an endophyte in oaks without showing pathogenic behavior [34]. This result suggests that other factors, such as *Armillaria*, *Phytophthora*, *Erysiphe alphitoides*, or abiotic stresses, may have contributed to the observed exudates. The first two species are typically observed on trees infested with beetles [35,36], particularly with the species *Q. suber* and *Q. ilex* [37,38]. In particular, *Phytophthora citricola*, *Phytophthora cinnamomi*, and *Phytophthora cambivora* lead to bleeding on the trunk of different oak species, typically occurring 1–2 m above ground level [10]. However, when alone, it does not seem to be able to trigger the decline of the tree directly [39]. Moreover, the severity of its impact varies considerably depending on site-specific environmental conditions [40].

## 3. Role of Other Biotic and Abiotic Factors in AOD Predisposition

**Figure 1:**
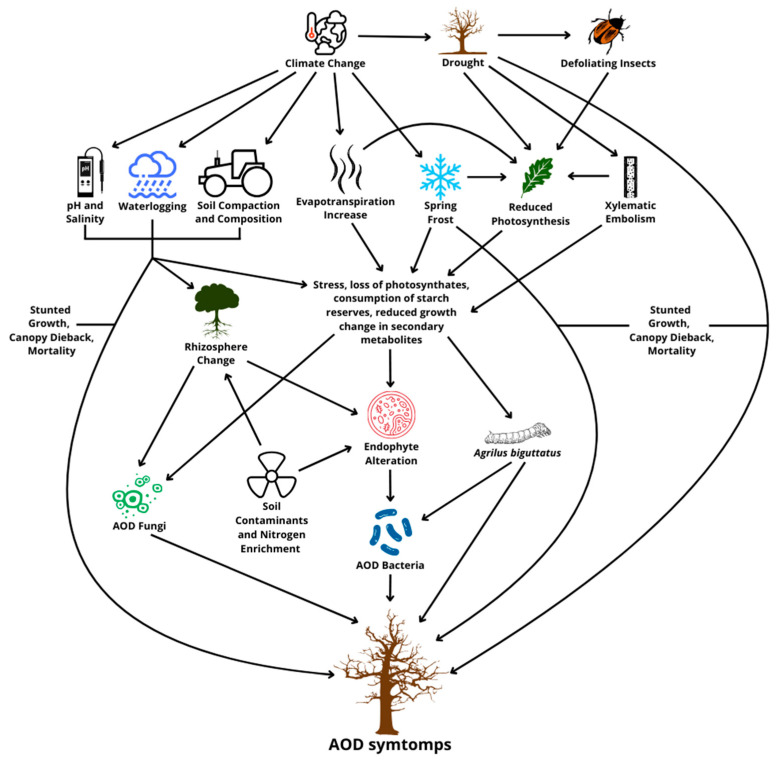
The biotic and abiotic factors involved in acute oak decline.

As described above, the decline of oaks is a complex phenomenon shared between several biotic and abiotic factors [41] (Figure 1). These factors may interact more or less directly, also creating cycles of positive effects that lead to the deterioration of the state of the plant in a short amount of time. Among the main predominant factors in the predisposition to decline, we find an increasingly recurring and current problem initiated by abiotic stress, such as drought, which provokes massive defoliation that increases the vulnerability of the oaks, supported by the role of herbivorous insects and attacks by wood-boring insect larvae, fungi, and bacteria [39]. Considered individually, abiotic stresses do not cause, in general, the entire decline and, therefore, the death of the plant; instead, they can induce significant physical, physiological, and chemical alterations. These changes, as mentioned, promote the proliferation of pathogens but also increase the attractiveness of trees for feeding and reproducing insects [42].

Defoliation in particular reduces the leaf surface, limiting the tree’s photosynthetic capacity. Among these insects, standout spring feeding species belonging to different families of Lepidoptera, such as Lymantriidae (*Lymantria dispar* L., *Euproctis chrysorrhoea* L.), Geometridae (*Operophthera brumata* L., *Erannis defoliaria* Cl.), and Tortricidae (*Tortrix viridana* L., *Archips xylosteana* L.) [3]. As a result, the development of new shoots to re-fold the crown is compromised, and the plant is exposed to carbon spills. In fact, the starch reserves of perennial organs remain depleted, reducing the oak’s tolerance to frosts [43] and slower radial growth [44]. As a result, trees particularly weakened by heavy defoliation are then more susceptible to other abiotic stresses [3]. Among the various stressors associated with AOD, the theme of prolonged drought, increasingly frequent due to climate change, has been examined [45]. In this regard, Ref. [46] found that warmer spring and summer temperatures increase evapotranspiration rates and, thus, the vulnerability of oaks [47]. Drought can also cause xylematic embolism, disrupting the tree’s plumbing system and eventually leading to foliage decay and branch mortality [4]. In addition, just like defoliation, drought-induced stress affects carbon allocation, making trees more susceptible to necrotrophic pathogens [48,49].

However, other environmental factors, such as late spring frost, flooding, and particularly cold winters, have been linked to reduced growth of oaks [50]. In parallel, soil composition can also have an impact, such as changes in characteristics such as land management, nitrogen deposition, heavy metal contamination, genetic predisposition, and changes in rhizosphere communities [15,39]. Finally, wood anatomy differences between oak species can influence their stress resilience [4]. Some soil properties, including pH and salinity, are thought to affect the composition of rhizosphere communities, shaping the oak microbiome according to the local pedoclimatic conditions [51]. Furthermore, Ref. [52] observed how the bacterial microbiome may be affected by changes in species based on tissue health, particularly by a higher concentration of Gram-positive bacteria in healthy tissues than of Gram-negative bacteria in diseased tissues [51]. High salinity levels can negatively affect water and nutrient absorption, limiting radial growth in oaks [53], while soils that are too compact and with reduced aeration may decrease root density [54]. It has been suggested that an excess of nitrogen in the soil accelerates growth to such a degree that there is potential for tree structural instability and water shortage if the root system cannot adequately support this expansion [55]. Nitrogen enrichment can also lower the allelochemical concentration of leaves, making trees more prone to insect attack [43]. Soil conservation treatments such as mulching may be effective in increasing the resilience of the oak system but require further investigation [56].

It has been noted that tree mortality rates increase with the increase in typical “D” exit holes in its bark, suggesting that the combined impact of *A. biguttatus* activity and existing lesions may contribute to the “acute” phase of AOD [57]. However, the specific role of the insect both as an accelerator of decline and as a likely vector for other pathogens remains uncertain. It is hypothesized that it could simply opportunistically exploit the weakened trees and not actively spread the phytopathogenic AOD [35]. In fact, the association between the beetle and bacteria can be random, with both benefiting independently from the stress condition of the oaks. As reported earlier, the coincidence of these two agents can trigger a positive cycle of reinforcement and increased stress, increasing the risk of death of the host [39].

## 4. Spatial Distribution of the Main Hosts and Bacteria

The genus *Quercus* is one of the most significant groups of woody angiosperms in the northern hemisphere, notable for its species diversity, ecological importance, and economic value. It comprises more than 600 species of trees and shrubs, which thrive in many habitats [58]. The predominant broadleaf species in Europe are *Q. robur* L. (pedunculate oak) and *Q. petraea* (Matt.) Liebl. (sessile oak). Their range begins in the north, from southern Norway and Sweden, and extends as far south as the northern Iberian Peninsula, southern Italy, the Balkans, and Turkey. *Q. robur* is a major player in timber production, so much so that it has also been introduced to the United States, where it has now naturalized in some regions [59]. At the same time, *Q. suber* and *Q. ilex* are also well adapted to the tropical and subtropical climates of Eurasia and North Africa [60]. This, therefore, highlights the risk of large-scale spread of AOD, finding favorable soil and climatic conditions in different continents and bringing significant environmental, social, and economic implications.

Oak decline has been documented in the UK and various parts of Europe over the past century [11], and bacteria have been frequently associated with different types of decline (Table 2). Ragazzi et al. [7] provide an overview of the earliest records of oak decline across Europe, linking specific oak species to affected countries. *Q. petraea* has been associated with cases in Germany (1739), Switzerland (1850), France (1921), Poland (1940), Austria (1944), the Czech Republic and Slovakia (1954), Hungary (1877), the former Yugoslavia (1878), Russia (1892), and Romania (1910). Similarly, *Q. robur* has been connected to decline events in Germany (1739), Switzerland (1850), Hungary (1877), the former Yugoslavia (1878), Romania (1910), Belgium (1921), France (1921), Poland (1940), Austria (1944), the Czech Republic and Slovakia (1954), Italy (1980), and Bulgaria (1982). Meanwhile, *Q. suber* has been linked to decline in Portugal (1988) and Spain (1989).

**Figure 2:**
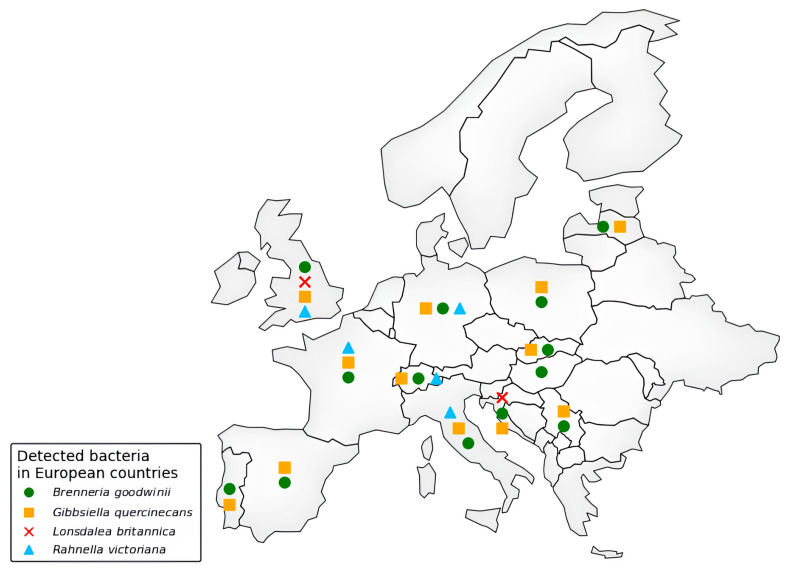
Distribution of AOD-associated bacteria in European countries (according to Scopus and EPPO databases, last access 17 March 2025).

Despite so many reports of the general condition of oak decline, even particularly dated ones, AOD was first formally identified in the UK only in 2014, affecting *Q. robur* and *Q. petraea* [21,61]. Indeed, Britain offers an ideal climate pool for the development of, in particular, the bacteria associated with AOD, including *B. goodwinii* [20], *G. quercinecans* [27], *R. victoriana* [52], and *L. britannica* [70]. In this regard, Ref. [71] points out that temperature is a key factor for bacterial proliferation, indicating a threshold value of 1.19 °C, below which such bacteria and possible vectors are restricted. However, the continuous increase in average global temperatures could widen the range in which these bacteria can thrive. Indeed, recent years have seen an outflow from Great Britain and a gradual expansion into various areas across Europe (Figure 2) and, beyond Europe, into countries such as Iran [19,72]. Samples of symptomatic exudates from oak trunks were collected and analyzed to investigate this decline. Real-time PCR confirmed the presence of the bacterial complex associated with AOD. In Switzerland, *B. goodwinii*, *G. quercinecans*, and *R. victoriana* were identified for the first time in 2017 on *Q. petraea*, showing stem bleeding symptoms [63]. Similarly, in June 2017, these bacteria were detected on *Q. robur* in Asturias, Spain [62]. Latvia also recorded its first occurrence of *B. goodwinii* and *G. quercinecans* in 2018, affecting multiple *Q. robur* forest sites [12]. In Portugal, AOD symptoms were observed in March 2018 on *Q. suber* in Alcácer [67]. Poland reported bacterial presence in 2019 when declining *Q. robur* trees in the Chojnów Forest District were found to host *B. goodwinii* and *G. quercinecans* [64]. AOD has also been observed in Iran’s Caspian Hyrcanian forests, with bacterial infections confirmed in 2020 [19,28]. In Germany, a study conducted by Julius Kühn-Institut [73] confirmed the first detection of *B. goodwinii*, *G. quercinecans*, and *R. victoriana* in the country; the same happened in France in 2024 [16]. In 2021, Pernek et al. [74] detected the presence of *B. goodwinii*, *G. quercinecans*, and *L. britannica* in Croatia. Tkaczyk et al. (2025) [75] detected *B. goodwinii* and *G. quercinecans* in Serbia, and these bacteria have also been reported in weakened *Q. robur* stands in Slovakia [66]. Likewise, in Salento, a coastal Mediterranean region in southern Italy, *Q. ilex* trees exhibiting AOD-like symptoms have tested positive for AOD-related bacterial infection [14].

Beyond Europe, *Brenneria* species have been isolated from the symptomatic bark of *Populus × euramericana* cankers in Henan Province, China, establishing no direct link to AOD [25]. In contrast, oak decline has also been attributed worldwide to fungal pathogens such as *Ophiostoma* spp. and *Phytophthora plurivora*, which are often associated with bark-boring insect activity in the Czech Republic [9]; *Erysiphe alphitoides*, which appears to contribute to a reduction in the leaf canopy, along with herbivorous insects and abiotic stresses in Poland [76]; Botryosphaeriaceae species, including *Diplodia* spp. in California [77]; and *Tubakia dryina*, which causes several leaf spot diseases [78].

## 5. Role of Agrilus Biguttatus in AOD

The interior of oak stems is beneficial to a wide range of insects, which vary according to the tree’s stage of decay [57]. Seven insect species, including *A. biguttatus*, *Coraebus florentinus*, *Coraebus undatus*, *Cerambyx cerdo*, *Platypus cylindrus, Scolytus intricatus*, and *Corythucha arcuata*, are frequently identified in AOD diagnostics in Europe and are often associated with different microbial communities [79,80]. Notably, the genus *Agrilus* comprises around 3000 species and subspecies [81], among which *A. biguttatus* F., *A. sulcicollis* Lac., *A. angustulus* Ill., and *A. viridis* L. are particularly significant. However, the insect most frequently associated with AOD is *A. Biguttatus* Fabricius, whose presence is often confirmed following cases of extensive defoliation or severe drought [80].

In Europe, *A. biguttatus* is considered one of the most significant secondary pests of oak trees [35], particularly those already weakened by abiotic and other stress factors [43,71]. It appears that stressed trees produce volatiles that attract these insects [80], as xylophagous species tend to target trees with weakened defenses, thereby accelerating their decline [39,57]. Mattson et al. [82] hypothesized that this attraction may also be due to the insect’s ability to detect drought-induced ultrasonic emissions caused by the breakage of water columns in the xylem of stressed trees. The beetles seem to prefer attacking areas of the stem beneath the crown, particularly those exposed to sunlight, and they prefer trees with thicker bark, which may provide better protection from parasitoid insects [83]. Its distribution could develop as a function of precisely these microhabitats [84].

Specifically in the context of AOD, *A. biguttatus* is consistently associated with different species of Gram-negative bacteria [27]. Indeed, where symptoms of this disease are observed, it is common to find the insect’s exit holes, characterized by their distinctive “D” shape [61,82]. Brown and co-workers [85] observed that the disease spread to neighboring trees in a clustered pattern, suggesting a biotic way to spread. However, the role of *A. biguttatus* as a vector for the bacterial agents of AOD has not yet been confirmed with sufficient evidence [39]. Females can lay up to 82 eggs deep within bark crevices, from which larvae emerge and progress through four instar stages before reaching adulthood [35]. The larvae feed on the inner bark, the cambial layer, and the outer sapwood, creating several inter-crossing galleries up to 1.5 m long, and later form chambers in the outer bark, where they overwinter and pupate [83]. Preferred sites for larval development include cavities, cracks in the wood, areas of bark loss exposing the sapwood, deadwood in the tree crown, and burls [86]. In the initial stages of colonization, the larvae could remain confined by the plant, causing very little damage. The plant’s health also influences its response to the larval attack, as it possesses specific defensive strategies, such as callus formation and gallery flooding [57]. These mechanisms support wound healing by enabling compartmentalization [80]. Consequently, *A. biguttatus* appears to visit weakened trees for oviposition significantly more often than healthy ones [57]. However, when there is a massive infestation within the wood, bark cracks are produced, the stem becomes deformed, and the plant eventually dies [43].

According to the interpretation of AOD symptomatology, larval excavation of the xylem disrupts the movement of water and mineral nutrients. At the same time, interference with the phloem hampers the transport of assimilates and hormones synthesized in the leaves. This disruption creates nutrient deficiencies and upsets the plant’s normal water status and carbon/nitrogen balance. Nevertheless, tree death only occurs in cases of high colonization (more than 50 larvae) [83]. Adults emerge from the wood two years after oviposition to feed and mate in the canopy [57]. The external symptoms caused by *A. biguttatus* also attract other insect species, fungi, and bacteria, triggering cellular post-mortem reactions. These reactions lead to bleeding dark exudations and tylosis formation in xylem vessels [83]. Notably, these include *B. goodwinii*, *G. quercinecans*, and *L. britannica*, though their relationship with the insect remains unclear. It is uncertain whether *A. biguttatus* acts as an accidental vector carrying these bacteria as part of its microbiota, whether the bacteria spread between neighboring trees independently, or whether they are merely endosymbionts that take advantage of plant decay following abiotic stress and subsequent insect attack [2]. Indeed, there is evidence of their co-occurrence in the majority of cases. Still, regarding this role as a vector, there has only been one study in this regard, in which Ref. [87] identified *G. quercinecans* within the gut of only one *A. biguttatus* insect out of 20 captures.

On the other hand, no evidence has been found concerning the potential transmission of the bacterium to a plant, so we cannot yet attribute this role to the insect. Regarding fungi, the beetle has been associated with the spread of *Collybia fusipes* and the presence of *Phytophthora* spp., for which it may serve as a potential vector [83]. Finally, assessing the influence of temperature on the insect’s development cycle and its potential for spread is particularly interesting [88]. In this regard, Ref. [35] warns of the likely expansion of the insect’s range and, thus, the potential increase of AOD in Europe as climate change progresses. Higher temperatures may facilitate its spread into areas previously unsuitable for its development [52,71].

Various approaches have been implemented to control or contain the insect and mitigate the impact of AOD on oak trees. One strategy involves eliminating the insect through chemical treatments applied to infested or trapped trees [89]. However, no biological control methods have been tested to date. Additionally, preventive strategies focus on enhancing the resilience and vigor of oak trees through targeted forestry practices [80].

## 6. Diagnostic Tools for AOD-Associated Bacteria

The genera or species of bacteria that can be found in oaks suffering from AOD have been observed. However, identifying them from samples taken in the field is not always easy. Sampling can be complex in terms of the removal of material to be used. Still, it is not always possible to find the presence of the bacteria hypothesized, as it could depend on their concentration, the developmental state of the pathology, or trivially on the portion of material removed from the tree. Generally, the sample consists of small sections picked from the edge of necrotic and healthy bark and sapwood tissues [28]. In any case, the identification of bacteria in plant pathology is carried out using various molecular biology techniques that may consider analysis from nucleic acids, proteins, or even volatile compounds [90]. The best performing technique is that based on polymerase chain reaction (Table 3), and in particular, techniques capable of obtaining amplification and analysis of several DNA or RNA target sequences simultaneously are currently used, such as multiplex PCR [26], HRM, DNA microarray [91], and DNA fingerprinting [13]. A second approach is related to the analysis of specific proteins linked to bacterial activity, studied through enzyme-linked immunosorbent assay, flow cytometry, or immune-fluorescence [92], or even the analysis of volatile organic compounds (VOCs) through gas chromatography–mass spectrometry [93] and electronic-nose devices [94] or through the creation of hyper-spectral images that can indicate changes in the state of the plant even in the primary stages of the disease, but these approaches are still used little and not as effective in the case of AOD.

In conclusion, there are also techniques based on the use of nanotechnology, particularly nanoparticles, such as array-based nano-sensors [95] or gold nanoparticles. Next comes the taxonomic classification of the pathogen identified. In particular, for prokaryotes, the analysis of the gene coding for 16S ribosomal RNA is widely used since it is highly conserved across bacterial species [96]. On the other hand, for specific genera such as Pseudomonas, it is recommended to use protein-coding genes such as gyrase beta subunit (gyrB), RNA polymerase 70 sigma factor (rpoD), and beta subunit of the RNA polymerase (rpoB), as they are less evolutionarily preserved [97]. The next and final step would then be the complete sequencing of the DNA of the specific localized bacterium, a strategy that is becoming increasingly more used and more affordable [90].

## 7. AOD Management Strategies

AOD is not currently listed in the EC Plant Health Directive nor on the EPPO Alert List, nor are the associated bacteria and their presumed vector, *A. biguttatus*. Only in the UK has this disease been listed in the Defra Plant Health Risk Register.

The eradication of AOD can be considered only in sites with a few impacted oaks. In this regard, good silvicultural practices are recommended to reduce the spread and impact of the disease. Management focuses on finding the causes and considering coordinated management actions, with no statutory measures against detected cases [98]. Silvicultural practices to limit predisposing factors such as drought or defoliation are necessary choices so that the disease does not spread and so recovery can be favored in already-affected trees. The removal and destruction of damaged bark or dead oaks due to AOD, preferably during scheduled selective thinning and not during rainy conditions, will limit the future emergence of damaging insects and their possible role as vectors of AOD-associated bacteria [61,84]. On the other hand, this choice is hotly debated. To date, it is preferable to cut down trees only if there are only a few affected individuals within a forest; otherwise, it is preferred to leave them. Pruning trees that are already infected is, on the other hand, not recommended at all [27]. Again, control of material that is moved from an infected to a non-infected area, such as firewood, should be organized and enforced [99].

Finally, another not insignificant possibility is to make the forest attractive to woodpeckers or parasitoid insects, favoring their population growth to prey on *A. biguttatus*, thus limiting the damage done to the bark of oak trees [100,101]. The application of insecticides is discussed, but they are only partially effective. In fact, it is noted that the application of pyrethroids can only control the insect in the outermost layer of the bark (in pupal chambers) but not the larvae, which feed deep down into the sapwood [102]. The use of systemic products has yielded more results, but their use is debated due to their impact on other species that inhabit oak wood, with related damage to the eco-systemic balance of forests [103].

Reforestation by planting new oak trees in areas affected by AOD can be considered, but with the caveat of increasing the biodiversity and thus the resilience of the affected species, rather than using only those that are already present in the area and particularly susceptible [104].

Certainly at the basis of controlling the spread of the disease is the construction of a database of the current prevalence of symptoms, bacteria, and insects associated with AOD for each country involved, and consequently, the possibility of creating predictive models of its future spread; monitoring is therefore fundamental [27]. Concerning places where AOD is not yet present, the option of stricter control on timber that is imported, especially from countries already harboring the disease, or the possible imposition of timber treatments with temperatures higher than 60 °C to eliminate the bacteria before distribution, should not be underestimated [27,96]. The possible active participation of ordinary citizens in helping to identify trees affected by AOD symptoms in forests close to urban centers should not be underestimated either, so it is good to inform and educate on the issue.

## 8. Conclusions

AOD is a relatively new phenomenon that is particularly complex, but its exact nature is unclear at present due to the co-participation of numerous abiotic and biotic factors. It has been pointed out that there are still several gaps in the general understanding of the disease, which increases the risk of AOD spreading undisturbed and control strategies being applied too late. In this sense, it is crucial to emphasize the importance of additional study in this field to better understand the disease’s causes and interactions, as well as the possibilities for control. In this regard, an interdisciplinary approach integrating ecology, microbiology, entomology, and forestry could prove crucial in addressing the complexity of AOD and identifying more effective solutions.

Furthermore, constant monitoring using advanced technologies, such as remote sensing, machine learning, and GIS systems, could significantly improve the ability to detect the disease early and develop predictive models that can anticipate its spread. In parallel, sustainable forest management, through tree species diversification, soil care, and the use of beneficial bio-inoculation, could contribute to increasing forest resilience and limiting the impact of AOD. Therefore, future studies should focus on analyzing the pathogenicity of the bacteria and their interaction with other species, the role of *A. biguttatus* in the transmission of AOD-associated bacteria, and the possible active spread of the disease, as well as the responsibility of fungal agents participating in the decline. In addition, strategies to increase the resilience of forests, which are still not considered, will need to be investigated.

## Figures and Tables

**Table 1:** Summary table describing the four AOD-associated bacteria.

Bacterium	Gram Stain	Characteristic	Role in AOD
*Brenneria goodwinii*	Gram-negative	Facultative anaerobe, necrotrophic behavior	Production of enzymes such as pectinases, cellulases, and tannins that degrade wood cell walls, secretion of virulence factors that promote necrosis
*Gibbsiella quercinecans*	Gram-negative	Facultative anaerobe, facultative necrotrophic behavior	Production of enzymes such as pectinases, cellulases, and tannins that degrade wood cell walls, secretion of virulence factors that promote necrosis
*Rahnella victoriana*	Gram-negative	Facultative anaerobe, opportunist behavior	Role not clear, probable synergy with *B. goodwinii* and *G. quercinecans*
*Lonsdalea britannica*	Gram-negative	Facultative anaerobe, facultative necrotrophic behavior	Role not clear, probable synergy with *B. goodwinii* and *G. quercinecans*

**Table 2:** Reported occurrences of AOD-associated bacteria and *Brenneria quercina* on various oak species (*Quercus* spp.) across different countries.

Pathogen	Host	Country	Reference
**AOD-associated bacteria**	*Q. robur*	Britain	[61]
		Spain	[62]
		Switzerland	[63]
		Poland	[64]
		Latvia	[65]
		Slovakia	[66]
	*Q. petraea*	Britain	[61]
		Switzerland	[63]
	*Q. suber*	Portugal	[67]
	*Q. ilex*	Spain	[62]
		Italy	[68]
	*Q. cerris*	Switzerland	[63]
	*Q. pubescens*	Switzerland	[63]
	*Q. rubra*	Switzerland	[63]
	*Q. pyrenaica*	Spain	[62]
***Brenneria quercina***	*Q. ilex*	Spain	[69]
	*Q. pyrenaica*	Spain	[69]

**Table 3:** Primers (5′-3′) employed for qPCR to detect bacteria associated with oak decline.

Bacteria	qPCR	Reference
*Brenneria goodwinii*	Bg99F: CTGGCCGAGCCTGGAAAC Bg179R: AGTTCAGGAAGGAGAGTTCGC FAM-CCAGAATCTCATATTCGAACTCCACCATGTT-BHQ1	[26]
*Gibbsiella quercinecans*	Gq284F: GGCTTTGATAGTGGTGGCC Gq418R: CGTTCCGTTATCACCGTGG Cy5-AACAGTTCCAGCGCCATTTTCTTCG-BHQ3	[26]
*Lonsdalea britannica*	Lq503F: GCAAGAAAGCCAAAATCAGC Lq634R: TCTTCACTTCGGACGACACC JOE-TGCTGTGGTATCGGTGAAAGTGCCC-BHQ1	[26]
*Rahnella victoriana*	Rv15F: CACCCAGACTTACGTGCAT Rv134R: TCAGTGTGATTGGTGAAGGT ROX-AGTGATTGGCGATACTGACGTGACC-BHQ2	[26]

## Data Availability

No new data were created or analyzed in this study.

## Abbreviations

The following abbreviations are used in this manuscript:

AOD	Acute oak decline
PCR	Polymerase chain reaction
HRM	High-resolution melting analysis
EPPO	European and Mediterranean Plant Protection Organization
VOCs	Volatile organic compounds
